# Blood Flow Restriction Resistance Exercise Improves Muscle Strength and Hemodynamics, but Not Vascular Function in Coronary Artery Disease Patients: A Pilot Randomized Controlled Trial

**DOI:** 10.3389/fphys.2019.00656

**Published:** 2019-06-12

**Authors:** Tim Kambič, Marko Novaković, Katja Tomažin, Vojko Strojnik, Borut Jug

**Affiliations:** ^1^Laboratory of Kinesiology, Faculty of Sport, University of Ljubljana, Ljubljana, Slovenia; ^2^Department of Vascular Diseases, Division of Internal Medicine, University Medical Centre Ljubljana, Ljubljana, Slovenia; ^3^Faculty of Medicine, University of Ljubljana, Ljubljana, Slovenia

**Keywords:** blood flow restriction, resistance training, low-loads, coronary artery disease, cardiac rehabilitation

## Abstract

Resistance training may be associated with unfavorable cardiovascular responses (such as hemodynamic alterations, anginal symptoms or ventricular arrhythmias). In healthy adults, blood flow-restricted (BFR) resistance training improves muscle strength and hypertrophy improvements at lower loads with minimal systemic cardiovascular adverse responses. The aim of this study was to assess the safety and efficacy of BFR resistance training in patients with coronary artery disease (CAD) compared to usual care. Patients with stable CAD were randomized to either 8 weeks of supervised biweekly BFR resistance training (30–40% 1RM unilateral knee extension) or usual exercise routine. At baseline and after 8 weeks, patients underwent 1-RM knee extension tests, ultrasonographic appraisal of *vastus lateralis* (VL) muscle diameter and of systemic (brachial artery) flow-mediated dilation, and determination of markers of inflammation (CD40 ligand and tumor necrosis factor alfa), and fasting glucose and insulin levels for homeostatic model assessment (HOMA). A total of 24 patients [12 per group, mean age 60 ± 2 years, 6 (25%) women] were included. No training-related adverse events were recorded. At baseline groups significantly differ in age (mean difference: 8.7 years, *p* < 0.001), systolic blood pressure (mean difference: 12.17 mmHg, *p* = 0.024) and in metabolic control [insulin (*p* = 0.014) and HOMA IR (*p* = 0.014)]. BFR-resistance training significantly increased muscle strength (1-RM, +8.96 kg, *p* < 0.001), and decreased systolic blood pressure (-6.77 mmHg; *p* = 0.030), whereas VL diameter (+0.09 cm, *p* = 0.096), brachial artery flow-mediated vasodilation (+1.55%; *p* = 0.079) and insulin sensitivity (HOMA IR change of 1.15, *p* = 0.079) did not improve significantly. Blood flow restricted resistance training is safe and associated with significant improvements in muscle strength, and may be therefore provided as an additional exercise option to aerobic exercise to improve skeletal muscle functioning in patients with CAD.

Clinical Trial Registration: www.ClinicalTrials.gov, identifier: NCT03087292.

## Introduction

Exercise training is a core component of the cardiac rehabilitation (Leon et al., 2005; Balady et al., 2007), with aerobic training recommended as the preferred modality (Bjarnason-Wehrens et al., 2004). Recommendations for resistance training have only recently and cautiously been put forward (Bjarnason-Wehrens et al., 2004). On the one hand, resistance training improves muscle strength, endurance and mass, bone density, and quality of life (Bjarnason-Wehrens et al., 2004; Williams et al., 2007; Wise and Patrick, 2011); while on the other hand, concerns have been raised over potentially unfavorable cardiovascular responses, such as blood pressure elevation, myocardial ischemia, and ventricular dysrhythmias (Haslam et al., 1988).

Current American Heart Association recommendations on resistance training in cardiovascular patients suggest lower loads [30% of one-repetition maximum (1-RM) for the upper limbs and 50–60% 1-RM for the lower limbs], as this would still improve muscle strength and endurance without excessive blood pressure elevation or other adverse cardiovascular events (Williams et al., 2007; Wise and Patrick, 2011). However, recent studies have shown some conflicting evidence against current resistance training guidelines in cardiovascular disease patients. One study has shown that moderate exercise loads (15 RM) induced greater hemodynamic response compared to higher exercise loads (4 RM) (Gjovaag et al., 2016), whereas others suggested longer sets may evoke higher hemodynamic drifts compared to shorter sets (Lamotte et al., 2010). Also, evidence suggests that higher training loads (>75% of 1-RM) are needed for optimal improvements in muscle hypertrophy and strength in healthy adults (American College of Sports Medicine [ACSM], 2009), whereas recommended loads for patients with coronary artery disease (CAD) are much lower (<30% 1-RM) (Bjarnason-Wehrens et al., 2004; Piepoli et al., 2011) and thus possibly insufficient to elicit increases in isometric strength and hypertrophy.

Blood flow restriction (BFR) exercise is a novel exercise modality in clinical settings, which induces muscle hypertrophy and strength with low to moderate training intensity through increased anabolic processes mediated by BFR (usually with cuff inflation) (Manini and Clark, 2009). BFR improves training adaptations (Loenneke and Pujol, 2009), such as muscle hypertrophy, muscle strength (Takarada et al., 2000; Madarame et al., 2008; Yasuda et al., 2014b), endurance (Kacin and Stražar, 2011) and acute hormonal responses (Pearson and Hussain, 2015), with minimal adverse cardiovascular or muscular effects (Manini and Clark, 2009). In healthy adults, BFR resistance exercise yields muscle hypertrophy and strength comparable to heavy-load resistance training (Hughes et al., 2017), using loads as low as 30% of 1-RM (Takarada et al., 2000). In addition to the improvement in muscle strength and hypertrophy, BFR resistance exercise was proven to be safe, with no significant differences following training in resting creatine kinase, interleukin-6, insulin-like growth factor 1 (IGF-1) or hemostatic markers (Fujita et al., 2007; Nakajima et al., 2007; Madarame et al., 2010; Karabulut et al., 2013; Patterson et al., 2013). Although most BFR research focused on muscular effects, it is important to note that resistance and aerobic BFR exercises may cause increases in heart rate and blood pressure that are greater than those observed with exercise performed at a similar intensity without BFR (Hackney et al., 2012). Since most BFR studies were conducted in healthy older adults (Karabulut et al., 2010, 2011; Yasuda et al., 2014b; Vechin et al., 2015) and musculoskeletal settings (ACL reconstruction, knee osteoarthritis) (Hughes et al., 2017), it is important to evaluate the cardiovascular response in individuals presenting with cardiovascular disease risk factors in a controlled settings (Hackney et al., 2012).

To date, only one study examined the acute effect of BFR resistance exercise in cardiovascular patients (Madarame et al., 2013). Apart from steadily increased heart rate, no adverse effects were reported during and after acute bouts of exercise, as the increase in haemostatic and inflammatory markers was independent of the exercise (Madarame et al., 2013). Chronic effects were observed in a recent trial in which decrease in brain natriuretic peptide and C-reactive protein were shown after 6 months of low intensity BFR aerobic cycling exercise in patients with chronic heart failure (Yasushi and Yudai, 2018), as the chronic impact of BFR resistance exercise in CAD patients still remains elusive. Therefore, we wanted to assess the impact of low-load BFR resistance training in patients with CAD on muscle strength and hypertrophy, vascular function, safety, cardiovascular responses, inflammatory markers, and insulin resistance.

## Materials and Methods

### Participants

Patients with stable CAD were recruited from the Center for Preventive Cardiology, Department of Vascular Diseases, Division of Internal Medicine, University Medical Centre Ljubljana, or Coronary Club Ljubljana, both located in Slovenia.

Patients with documented CAD (>3 months after a myocardial infarction, percutaneous coronary intervention or/and coronary artery bypass grafting), aged between 18 to 75 years and physically active more than three times a week were included into the study. All participants were clinically stable and engaged in regular unsupervised physical activity (e.g., walking, cycling) after completion of cardiac rehabilitation, as assessed with short interview. Exclusion criteria were unstable or uncontrolled cardiovascular disease/event (unstable angina, recent myocardial infarction <3 months prior to inclusion, class III or IV heart failure, uncontrolled dysrhythmias, severe pulmonary hypertension, severe and/or symptomatic valve disease, acute myocarditis, endocarditis, or pericarditis, aortic syndrome or venous thromboembolism), acute systemic illness, uncontrolled hypertension (>180/110 mmHg), postural hypotension (≥20 mmHg drop in systolic blood pressure with symptoms of dizziness or light-headedness) (Williams et al., 2007; Wise and Patrick, 2011).

### Study Design

**FIGURE 1:**
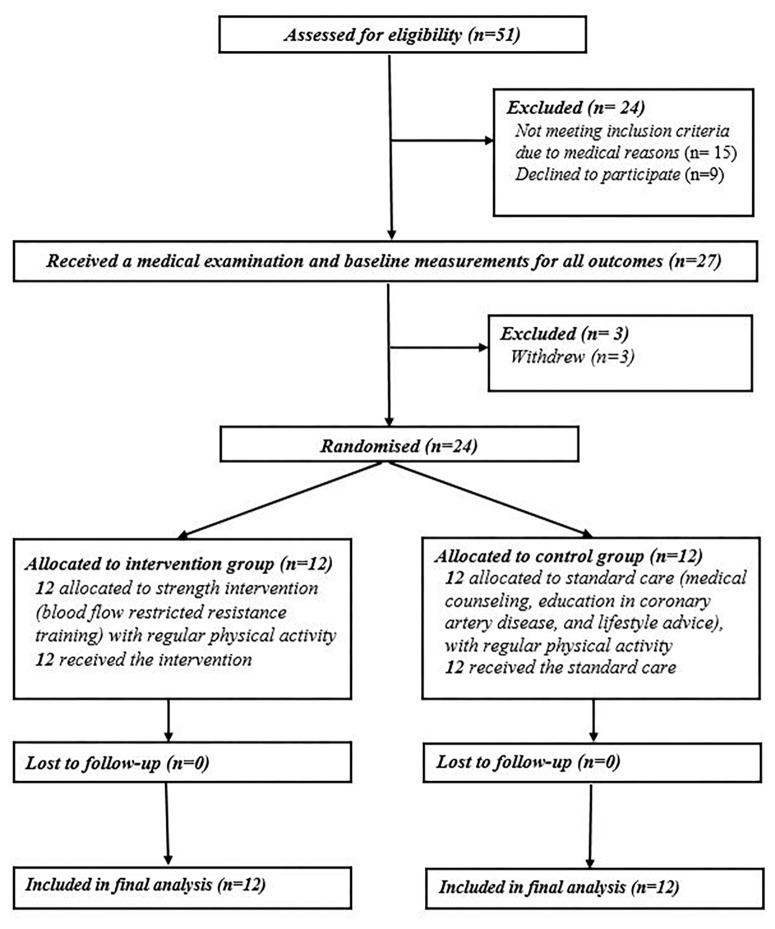
Flow diagram of parallel randomization.

Study was designed as randomized, open-label clinical trial aligned with the CONSORT guidelines (Schulz et al., 2010), adapted for two parallel groups (Figure 1). Participants were randomized into 2 groups (1 interventional and 1 control group) with ratio of 1:1 using adaptive (urn) randomization with sealed envelopes and randomization concealment from the recruiting investigator. Measurements were performed two times: at baseline and after the intervention period (8 weeks). Patients in both groups underwent clinical examination prior to inclusion and ultrasonographic assessment [systemic brachial vascular function and muscle thickness of m. *vastus lateralis* (VL)], muscle strength assessment and blood sample withdrawal at baseline and post-training intervention. The primary outcome of the study was change in muscle strength and hypertrophy from baseline to 8-week follow-up. The secondary outcome was change in vascular function. Other exploratory outcomes included cardiovascular markers, insulin resistance and inflammatory markers.

Measurements and exercise intervention were conducted at the Center for Preventive Cardiology, University Medical Centre Ljubljana, Slovenia. One week ahead of baseline measurement participants were familiarized with testing and training procedures. The intervention period lasted for 8 weeks of BFR resistance training period with baseline and post-training testing.

During the week ahead of study, all subject underwent full medical examination and a familiarization session. Each testing session was split to 2 days, with at least a day of rest between testing. On the first day, ultrasonographic images were taken to assess flow mediated dilatation (FMD) and muscle thickness, and unilateral 1-RM strength was assessed as well. During the second day, blood samples were collected before subjects performed 3 sets of 10 repetitions of BFR resistance exercise at an intensity of 30% of their previously achieved 1-RM leg extension. All subjects performed a standardized warm-up before each testing or training session as noted in previous section.

Prior to inclusion, all patients underwent a run-in phase aerobic exercise training for 4 weeks (3 times a week, 45 min of cycling, walking or combination thereof at 70–80% maximum heart rate). After the baseline measurements, the control group continued with usual care (aerobic exercise training), while in the intervention group BFR training was added on usual care.

Both groups were informed of the risk associated with the methods and procedures, and signed written consent prior to their inclusion. The protocol was approved by the Republic of Slovenia National Medical Ethics Committee and was registered on ClinicalTrials.gov (identifier: NCT03087292). The study was conducted in accordance with the Declaration of Helsinki and the American College of Sports Medicine guidelines for Use of Human Participants.

### Exercise Intervention

Subjects in BFR resistance training group trained for 8 weeks, performing a total of 16 unilateral leg extension exercise sessions. During each week two exercise sessions were performed with 48 h of rest period in between. Each training sessions consisted of three sets of 8, 10, and 12 repetitions in first, second, and third set, respectively, with 45 s inter-set rest interval. Training intensity was initially set to 30% of 1-RM. At every next training session the number of repetitions was increased for at least two repetitions per set. Additionally, training load was increased every 2 weeks, from initial 30% 1-RM to 32.5% 1-RM in the third week, to 37.5% 1-RM in the fifth week and finally to 40% 1-RM in the seventh week. Furthermore, as the training intensity increased, the number of repetitions was lowered to those in first training session. A lifting cadence of 1 s:2 s (concentric: eccentric part) was used for both groups throughout the full range of motion. For exercise purposes, BFR was applied by compressing the medium part of each thigh separately using a pneumatic cuff (Riester, Jungingen, Germany). The cuff was 23 cm width and 42–50 cm in length. Before each session or test, the cuff was inflated between 15 and 20 mmHg greater than resting brachial systolic pressure (Manini and Clark, 2009), taking into consideration the width of the cuff (Hackney et al., 2012) and thigh circumference (Loenneke et al., 2012a). Pressure was maintained throughout the entire training session and was released at the end of last set. To assure safety of the patients, brachial blood pressure and heart rate were measured at the rest, after sets and 5 min after the end of each training session. Brachial systolic and diastolic pressures were monitored using automatic blood pressure monitor (Omron M6, Omron Healthcare, Inc., Vernon Hills, IL, United States) and heart rate was obtained using telemetry (Polar, Kempele, Finland). All measurement and training sessions were monitored by cardiologist and kinesiologist.

### Maximal Muscle Strength Measurement

BFR resistance training and unilateral isotonic leg extension strength measurements were performed on leg extension machine (Technogym, Cesena, Italy) following previous recommendations (Brown and Weir, 2001; Karabulut et al., 2011). Maximal strength was assessed by performing 1-RM testing at baseline (pre-training) and after (post-training) the completion of exercise intervention. The participants were familiarized with exercise testing protocol and were advised with proper lifting technique at least 3 days prior to testing. Patients were advised to perform the exercise while seated in an upright position with their back in permanent contact with the machine during the test, and with hands holding the handles of the machine. Before the measurement, participants were instructed to complete a warm up that included a 5–8 min of brisk walking (>4 km/h) on treadmill followed by two sets of static stretching for quadriceps muscles. During the test, participants were instructed to complete a warm-up set comprised of 8–10 repetitions for each leg at approximately 50% of their perceived maximal effort (1-RM). The weight was then increased progressively each set with simultaneously lowering number of repetitions until reaching the maximum weight that could be lifted for one repetition. Between each maximal effort there was a 2–3 min rest interval. Maximal muscle strength was determined within five attempts. Before and after measurement brachial blood pressure was obtained, while heart rate was monitored throughout the procedure.

### Muscle Thickness and Vascular Function Measurement

Muscle thickness and vascular function was assessed with Aloka Prosound α7 ultrasound machine (Hitachi Healthcare Americas, Twinsburg, OH, United States) at baseline and after the completion of the study. Both measurements were performed at the same exact time of the day, mostly in the mornings.

Muscle thickness was assessed at rest, with subjects performing no physical activity at least a day before the testing. After applying hypoallergenic, watersoluble transmission gel, ultrasound transducer was placed on the surface of the skin. Muscle thicknesses of right VL were assessed. The measurement sites were determined at upper, midway and lower thirds between the greater trochanter and the lateral epicondyle of the knee. Those distances were measured with the subjects standing still with their knees fully extended. Three longitudinal images of the VL were recorded for each measurement, and the mean of the three values was used for further analysis (Martín-Hernández et al., 2013). Images were then independently analyzed by two experienced researchers and the mean of both was used as a final result.

Flow-mediated dilation was measured on the right brachial artery, approximately 5 cm above the antecubital fossa, according to previously described guidelines (Corretti et al., 2002). The participants were instructed to lie in supine position. The artery was firstly visualized in the horizontal position on the screen, after which three measurements of the arterial diameter were obtained (d1). A cuff was then inflated below the antecubital fossa with the pressure of 50 mmHg above the systolic blood pressure. Ischemia was maintained for 4.5 min. Sixty seconds after the cuff deflation, three measurements of the arterial diameter were obtained again (d2). FMD was calculated with the following formula: [mean(d2) - mean(d1)]/mean(d1) and expressed in %.

### Blood Markers Measurement

Blood samples were drawn from the antecubital vein using a 21-gauge needle; firstly, into a 4.5 mL vacuum tube containing 0.11 mol/L sodium citrate (9:1 v/v) (Becton Dickinson, Vacutainer System Europe, Heidelberg, Germany), followed by serum and K3-EDTA vacuum tubes (Laboratory Technic Burnik, Ljubljana, Slovenia). Plasma was prepared with 20-min centrifugation at 2000 ×*g* and 15°C. Serum was prepared with 20-min centrifugation at 2000 ×*g* and 20°C. Half milliliter aliquots of serum were snap frozen in liquid nitrogen and stored at ≤-70°C until analysis. Concentration of glucose was measured in fresh serum on the Fusion 5.1 biochemistry analyzer (Ortho Clinical Diagnostics, Rochester, NY, United States). Levels of CD40 ligand, insulin, TNF-α were measured in a thawed serum aliquote with the Luminex’s xMAP^®^ Technology utilizing magnetic beads coupled with specific antibodies, which allowed multiplexing. Analysis was performed according to manufacturer’s instructions (R&D Systems, Abingdon, United Kingdom). Homeostatic model assessment (HOMA) was calculated using values plasma glucose levels and insulin levels via the HOMA Calculator^1^.

### Sample Size Calculation and Statistical Analysis

Sample size was calculated based on muscle strength as our primary outcome, as the loss of muscle strength in CAD patients is attributed to long term bed confinement, physical inactivity and in some cases also significant impairment in the cardiovascular disease itself (Bjarnason-Wehrens et al., 2004). The calculations suggested that 28 patients with CAD should be included in order to detect effect size value for muscle strength after BFR training larger 0.58 as described previously (Loenneke et al., 2012b), with an actual power of 0.95 at a level of statistical significance <0.05. Statistical power and sample size was calculated using G^∗^Power statistical software (University of Düsseldorf, Germany).

Numeric variables were described as mean values and standard errors of mean, and categorical variables were described as numbers. Data were firstly screened for normality of distribution and homogeneity of variances and/or regression through Shapiro–Wilk’s test, Levene’s test and interaction between independent variables × covariate, respectively. Baseline and post-training differences between groups were determined with the Independent-Samples *t*-test for normally distributed variables and equal variances between groups, and Mann–Whitney *U* test was used for asymmetrically distributed variables and/or unequal variances between groups. Two-way analysis of variance (ANOVA) for repeated measurements was used to calculate main effects of group, time and group × time interaction. Within group effect of training intervention (baseline vs. post-training) was assessed with Paired-Samples *t*-test for normally distributed variables and with Wilcoxon’s test for asymmetrically distributed variables. All data are displayed in the text, tables and figures. The data were analyzed using IBM SPSS Statistics v.21 statistical software package for Windows (SPSS Inc., Chicago, IL, United States). The level of significance was set *a priori* to an alpha of <0.05.

## Results

**FIGURE 2:**
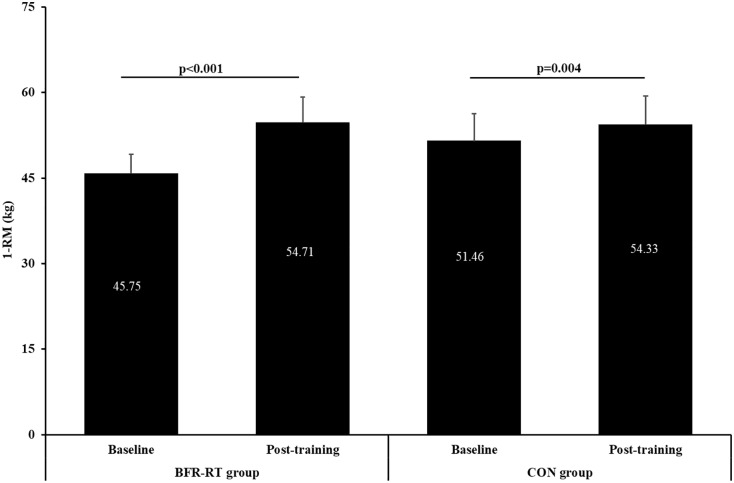
Baseline and post-training values of one repetition maximum (1-RM) for both groups. BFR-RT group, blood flow restriction-resistance training group; CON group, control group. Values are presented as mean ± SE.

During the recruitment process, 51 volunteers agreed to participate in the study. After initial medical examination and measurements, 24 were included into the study (6 women, 18 men; age 60.5 ± 2.4 years). Among excluded participants, 15 were excluded due to medical exclusion criteria and 12 left the study prior to start due to personal reasons/preferences (Figure 1). Patients were randomly assigned to either BFR resistance training group (3 women, 9 men; age 64.9 ± 1.6 years) or the control (CON) group (3 women, 9 men; age 56.2 ± 2.5 years) (Figure 2).

Among 24 included patients, all have completed the study (Figure 1). The adherence rate remained complete (100%) throughout the exercise intervention in both groups. Despite some occasional reports of muscle pain at the end of training, no deaths, adverse effect of exercise, such as muscle damage, skeletal injuries, chest pain, shortness of breath, dizziness, palpitations, venous thrombosis, pulmonary embolism or rhabdomyolysis, were reported.

Baseline physical and clinical characteristics of the sample and both groups are displayed in Table 1. Overall, there were significant differences in mean age (BFR resistance training group 64.9 ± 1.6 years vs. CON group 56.2 ± 2.5 years) and systolic blood pressure (BFR resistance training group 129.67 ± 3.71 mmHg vs. CON group 117.50 ± 3.36 mmHg), otherwise there were no significant differences between groups in other physical and clinical variables.

**Table 1:** Baseline characteristics.

	Sample (*n* = 24)	BFR-RT group (*n* = 12)	CON group (*n* = 12)	*p*
Age (years)	60.5 (2.4)	64.9 (1.6)	56.2 (2.5)	<0.001
Female/male ratio (N)	18/6	9/3	9/3	1.000
Height (cm)	172.30 (2.42)	169.53 (1.87)	175.08 (2.71)	0.106
Weight (kg)	86.78 (3.53)	86.55 (3.76)	87.01 (3.45)	0.929
BMI (kg/m^2^)	29.26 (1.11)	30.15 (1.25)	28.37 (0.94)	0.268
Systolic BP (mmHg)	123.58 (3.89)	129.67 (3.71)	117.50 (3.36)	0.024
Diastolic BP (mmHg)	80.04 (1.69)	81.92 (1.75)	78.17 (1.50)	0.118
Resting heart rate (bpm)	64.17 (3.15)	63.58 (2.68)	64.75 (3.67)	0.800
LVEF (%)	64.38 (1.43)	62.75 (1.69)	66.00 (2.28)	0.264
Post-surgery (years)	4.48 (0.83)	4.79 (1.30)	4.17 (1.09)	0.876
**Myocardial infarction**				
NSTEMI, N (%)	13 (54.2)	6 (50.0)	7 (58.3)	0.682
STEMI, N (%)	11 (45.8)	6 (50.0)	5 (41.7)	
**Surgical intervention**				
CABG, N (%)	5 (20.8)	2 (16.7)	3 (25.0)	1.000
PCI, N (%)	19 (79.2)	10 (83.3)	9 (75.0)	
**Medications**				
Aspirin, N (%)	24 (100.0)	12 (50.0)	12 (50.0)	/
Statin, N (%)	24 (100.0)	12 (50.0)	12 (50.0)	/
Beta blocker, N (%)	16 (66.7)	8 (50.0)	8 (50.0)	1.000
ACE/ARB, N (%)	17 (70.8)	8 (47.1)	9 (52.9)	1.000
**Cardiovascular risk factors**				
Arterial hypertension, N (%)	17 (70.8)	8 (47.1)	9 (52.9)	1.000
Hyperlipidemia, N (%)	23 (95.8)	11 (47.8)	12 (52.2)	1.000
Diabetes Mellitus, N (%)	5 (20.8)	3 (60.0)	2 (40.0)	1.000
**Smoking**				
Non-smoker, N (%)	7 (29.2)	3 (42.9)	4 (57.1)
Smoker, N (%)	4 (16.6)	2 (50.0)	2 (50.0)	0.822
Ex-smoker, N (%)	13 (54.2)	7 (53.8)	6 (46.2)	

*Data for continuous values are expressed as mean (±SE). BFR-RT, blood flow restriction-resistance training; CON, control group; BMI, body mass index; BP, blood pressure; LVEF, left ventricular ejection fraction; STEMI, ST, elevated myocardial infarction; NSTEMI, non ST-elevated myocardial infarction; CABG, coronary artery bypass graft; PCI, percutaneous coronary intervention.*

### Hemodynamic Response

Post-training responses of heart rate (RR), systolic and diastolic blood pressure following exercise intervention are shown in Table 2. At baseline there was a significantly lower systolic blood pressure in CON group (*p* = 0.024), with no significant differences in resting heart rate or diastolic blood pressure. After the training intervention, there was a significant main effect for group × time interaction (*p* = 0.001; η^2^ = 0.627) and trend for group effect (*p* = 0.074) on systolic blood pressure. Contrary, no significant main effects of time, group or group × time interaction for resting heart rate and diastolic blood pressure were observed. Furthermore, there was a significant decrease in systolic blood pressure in BFR group after training intervention (*p* = 0.030), whereas similar decrease was not observed in diastolic blood pressure and resting heart rate.

**Table 2:** Resting hemodynamics at baseline and after the intervention period.

Variable (unit)	Group	Baseline	Post-training	*p*
RR (bpm)	BFR-RT group	63.58 (2.68)	62.25 (1.88)	0.537
	CON group	64.75 (3.67)	63.67 (3.18)	0.729
Systolic BP (mmHg)	BFR-RT group	129.67 (3.71)	122.9 (2.74)	0.030
	CON group	117.50^∗^ (3.36)	120.08 (3.54)	0.306
Diastolic BP (mmHg)	BFR-RT group	81.92 (1.75)	79.67 (1.99)	0.133
	CON group	78.17 (1.50)	76.83 (3.15)	0.632


*^∗^Significantly different (p < 0.05) from BFR-RT group. Data are presented as mean (±SE). BFR-RT, blood flow restriction-resistance training; CON, control; RR, resting heart rate; BP, blood pressure.*

Acute hemodynamic responses to exercise are presented in Table 3. In both groups BFR exercise evoked significant increase in heart rate (*p* < 0.001) and systolic blood pressure at baseline and post-training exercise test, while diastolic blood pressure did not increase significantly. There was no significant difference between groups in hemodynamic change (at rest vs. post-last set).

**Table 3:** Acute hemodynamic exercise response at baseline and after the intervention period.

Variable (unit)	Group	Baseline		Post-training		Baseline Δ (*p*)	Post-training Δ (*p*)
		Pre exercise	Post-last set	Pre exercise	Post-last set		
RR (bpm)	BFR-RT group	63.58 (2.68)	81.75 (2.65)	62.09 (2.06)	83.46 (3.74)	18.17 (0.000)	21.36 (0.000)
	CON group	64.40 (4.39)	76.30 (3.97)	63.67 (3.19)	78.42 (4.07)	11.90 (0.000)	14.75 (0.000)
Systolic BP (mmHg)	BFR-RT group	131.46 (3.56)	143.14 (3.86)	122.37 (2.95)	142.82 (4.30)	11.68 (0.001)	20.45 (0.000)
	CON group	116.70 (3.98)	129.25 (2.11)	120.73 (3.82)	129.50 (5.80)	12.55 (0.014)	8.77 (0.035)
Diastolic BP (mmHg)	BFR-RT group	82.73 (1.70)	85.59 (2.16)	79.18 (2.12)	82.09 (2.31)	2.86 (0.112)	2.90 (0.217)
	CON group	77.50 (1.60)	80.25 (2.61)	77.09 (3.44)	78.64 (3.02)	2.75 (0.314)	1.55 (0.722)


*Data are presented as mean (±SE). RR, resting heart rate; BP, blood pressure.*

### Maximal Muscle Strength

At baseline and post-training there were no significant differences between groups in maximal muscle strength (Figure 2). After the training period, there was a significant main effect for time (*p* < 0.001, η^2^ = 0.769) and group × time interaction (*p* < 0.001; η^2^ = 0.724), but no for group (*p* = 0.233). Both groups significantly improved muscle strength post-training, with greater increase in the BFR resistance training group (16.37%, *p* < 0.001) than control group (5.29%, *p* < 0.01). Post-training leg strength increased for 8.96 and 2.88 kg in the BFR resistance training group and the control group, respectively.

### Muscle Thickness

VL muscle thickness was assessed pre- and post-training period (Table 4). There was no between-group difference in muscle thickness observed at baseline nor after the completion of the study. After the training intervention, there was a significant main effect for time in lower third of VL (*p* < 0.05; η^2^ = 0.360) alongside borderline significant time effect in midway third of VL (*p* = 0.072; η^2^ = 0.265), whereas no significant group × time interaction was obtained in neither of all three thirds of VL. Additionally, a significant post-training decrease in muscle thickness of lower VT was observed in CON group (*p* = 0.031). On the contrary, improvements of muscle thickness on upper and midway thirds of VL in the BFR resistance training group showed a trend toward statistical significance (1.58–1.67 cm, *p* = 0.096 and 1.60–1.68 cm, *p* = 0.082, respectively).

**Table 4:** Muscle thickness and blood markers at baseline and after the intervention period.

Variable (unit)		Baseline	Post-training	*p*
Upper third of VL diameter (cm)	BFR-RT group	1.58 (0.07)	1.67 (0.06)	0.096
	CON group	1.56 (0.05)	1.51 (0.07)	0.367
Midway third of VL diameter (cm)	BFR-RT group	1.60 (0.08)	1.68 (0.08)	0.082
	CON group	1.58 (0.05)	1.64 (0.06)	0.331
Lower third of VL diameter (cm)	BFR-RT group	1.64 (0.07)	1.57 (0.09)	0.429
	CON group	1.53 (0.07)	1.43 (0.08)	<0.05
Insulin (pg/mL)	BFR-RT group	1247 (132)	864 (180)	0.077
	CON group	655 (119)*	810 (253)	0.791
HOMA IR	BFR-RT group	4.01 (0.43)	2.86 (0.57)	0.079
	CON group	2.17 (0.40)*	2.38 (0.59)	0.733
CD40 ligand (pg/mL)	BFR-RT group	9116 (1100)	5735 (1221)	0.052
	CON group	7869 (1142)	5142 (784)	0.060
TNF-α (pg/mL)	BFR-RT group	5.8 (1.85)	4.9 (1.41)	0.581
	CON group	14.9 (6.41)	14.5 (4.99)	0.967


*Significantly different (p < 0.05) from BFR-RT group. Data are presented as mean (±SE). VL-m, vastus lateralis; HOMA, homeostatic model assessment; IR, insulin resistance; TNF-α, tumor necrosis factor alpha.

### Vascular Function

**FIGURE 3:**
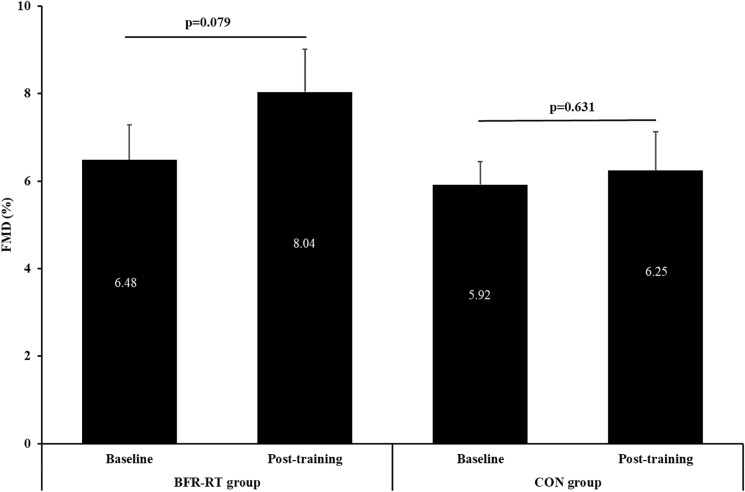
Baseline and post-training values of flow-mediated vasodilation (FMD). BFR-RT group, blood flow restriction-resistance training group; CON group, control group. Values are presented as mean ± SE.

Two-way ANOVA showed no significant main effect for group (*p* = 0.134) or group × time interaction (*p* = 0.28), whereas a borderline significance was observed for time effect (*p* = 0.092; η^2^ = 0.236). There were no significant differences between groups or pre- vs. post-training within group, although a trend toward improvement of FMD was obtained in the BFR resistance training group (6.48 ± 0.80% to 8.04 ± 0.98%, *p* = 0.079, respectively) (Figure 3).

### Blood Markers

Baseline between group differences were observed in insulin levels and insulin resistance (HOMA IR; both biomarkers *p* = 0.014; Table 4). After the training intervention, there were significant main effects for time (*p* = 0.036; η^2^ = 0.340) for CD40 ligand, significant main effect for group (*p* = 0.005; η^2^ = 0.306) and group × time interaction (*p* = 0.036; η^2^ = 0.342) for insulin levels. In BFR group, training intervention led to a borderline significant decrease in insulin (*p* = 0.077), HOMA IR (*p* = 0.079) and CD40 ligand levels (*p* = 0.052), while a similar trend was additionally observed in the control group for CD40 ligand levels (*p* = 0.060).

## Discussion

To our knowledge, this is the first study to investigate the beyond-acute effects of BFR resistance training with low-loads (30–40% 1-RM) in CAD patients. Exercise training proved to be safe in patients, and was associated with increased muscle strength and a trend toward increased muscle thickness and altered inflammatory response.

Our findings confirmed that 8 weeks of BFR resistance training may improve muscle strength as appraised by unilateral leg extension 1-RM. Previous studies have shown similar increases in leg extension 1-RM strength in comparable age groups of healthy individuals (Karabulut et al., 2010, 2011; Yasuda et al., 2014a,b), with the exception of one study (Vechin et al., 2015). Also, the magnitude of strength gains in our study is consistent with previous reports (in the range between 15 and 30%). However, longer training intervention (Yasuda et al., 2014b) or higher frequency and repetitions (Karabulut et al., 2011) seem to provide additional strength gains. In addition to muscle strength, our study also showed a trend in increased muscle thickness (as observed in upper and midway section of muscle *VL*). Conversely, previous studies in healthy students (Abe et al., 2005; Fujita et al., 2008; Madarame et al., 2008) and older adults (Yasuda et al., 2014a,b; Vechin et al., 2015) reported a definite increase in muscle hypertrophy of *VL* after BFR resistance training, which may be due to higher intensity, volume or duration of exercise trainings, as well as (younger) age of participants in these studies.

Resistance training under BFR reduced resting systolic blood pressure after 8 weeks in intervention group. This may be a result of lowered hemodynamic stress for a given muscle force after resistance training and less evoked rate of heart rate-pressure product (HR times SBP, an indirect index of myocardial oxygen demand) (Williams et al., 2007). Conversely, 12 weeks of whole body resistance training at 60–80% of 1-RM without occlusion did not evoke any hemodynamic response at rest in CAD patients (Grafe et al., 2018). Thus it may be postulated that BFR using lower loads could promote better training adaptations compared to higher loads resistance training without occlusion (Hackney et al., 2012). Our acute hemodynamic response to exercise is in line with previous reports in healthy older men (Staunton et al., 2015) and women (Scott et al., 2018) using BFR-RT, despite lower increase in heart rate. This can be explained with the discrepancies between exercise modes. In both studies participants performed leg press exercise training under BFR, which involve much more muscle mass than unilateral knee extension and thus may lead to higher hemodynamic response. Also, longer time under occlusion may evoke higher hemodynamic response, as both previous studies performed longer sets with at least 15 repetitions per set (Staunton et al., 2015; Scott et al., 2018).

The majority of previous studies in CAD have shown improvements in FMD after aerobic (Edwards et al., 2004; Blumenthal et al., 2005) or combined aerobic-and-resistance training (Vona et al., 2009; Anagnostakou et al., 2014). The vascular response, however, was higher with high intensity interval training (Ramos et al., 2015) or combined exercise training (Vona et al., 2009; Anagnostakou et al., 2014). Most clinical trials have shown improvements in FMD (Vona et al., 2009; Anagnostakou et al., 2014) after moderate resistance training, which is in line with our results. Apart from exercise type, the magnitude of effect is predominately associated with training duration, frequency and intensity, as longer interventions (>12 weeks), higher frequencies (3–4 times a week), higher intensities (>55% 1 RM) structured in whole body resistance regimens have the potential to provoke higher FMD response (Vona et al., 2009; Anagnostakou et al., 2014). Hence, discrepancies in training parameters may explain the modest FMD improvements in our study.

Inflammatory mediators appear to play a fundamental role in the initiation, progression, and eventual rupture of atherosclerotic plaques (Szmitko et al., 2003) and pathogenesis of cardiovascular diseases (Pedersen, 2017). Evidence suggests that increased TNF-α (Szmitko et al., 2003; Pedersen, 2017) and CD-40 ligand are linked to endothelial dysfunction and subsequent atherogenesis with late thrombotic complications (Szmitko et al., 2003). In contrast, physical activity can counteract with provoking anti-inflammatory effects by an inhibition of TNF-α (Pedersen, 2017) and CD-40 ligand levels (Bjornstad et al., 2008), although the latter mechanism was not proven following our BFR resistance training. The difference may occur as a result of short exercise duration (<15 min) and relatively limited muscle mass involved during the exercise (unilateral knee extension), as more pronounced systemic response between TNF-α and IL-6 were observed after longer strenuous exercise involving several large muscle groups (Pedersen, 2017). On the other hand, a 20-week combined endurance and resistance training decreased values of soluble CD-40 ligand in patients with chronic heart failure (Bjornstad et al., 2008).

BFR resistance training improved insulin sensitivity. Resistance training without BFR promotes a decrease in resting insulin levels and increase in insulin sensitivity (Williams et al., 2007), which is in line with our results. Similar favorable effects of resistance exercise with higher loads were also reported in patients with type 2 diabetes mellitus (Jorge et al., 2011; Kadoglou et al., 2013). Data from two previous study suggest that the decrease in HOMA IR can be time dependent, as there were no significant changes in HOMA IR after 12 weeks of resistance training in patients with type 2 diabetes mellitus of comparable age (Jorge et al., 2011). Contrary, a significant decrease in HOMA IR was obtained after 6 months of resistance training at the exercise intensity of 60–80% of 1-RM in patients with type 2 diabetes (Kadoglou et al., 2013). Furthermore, it is plausible that younger control group was more physically active prior to the documented coronary event, as this could explain their the significant lower baseline values of insulin and lower HOMA IR (Gayoso-Diz et al., 2011).

Our study has some limitations. Firstly, a relatively small sample size. This was reflected in a significant age-difference between intervention groups suggesting randomization failure, which possibly yielded pronounced between-group differences because of faster recovery in younger participants. Thus, our study was underpowered and should be regarded as pilot and hypothesis generating. Secondly, the allocation was not blind. The majority of subjects were physically active, which may have diminished the between-group differences and effects of our intervention itself. Moreover, as all patients (including control group) were made aware of positive effects of exercise on their strength and health in general, an additional increase in physical activity level during the study period cannot be ruled out. Thirdly, duration of the trial may have been too short to express sufficient responses. Since safety of BFR training has not been rigorously tested in clinical settings, and exact cardiovascular and coagulation responses were not clearly presented to this date, the duration of exercise intervention was chosen based on previous resistance training research in CAD patients. Nevertheless, longer exercise interventions (>12 weeks) might have provoked additional muscle gains and vascular function improvements. Lastly, virtually all patients were on secondary preventive medications, which may have confounded heart rate (beta-blockers), and blood pressure and vascular function (statins and ACE-inhibitors).

Our results have shown that BFR resistance training is efficient only in terms of improving muscle strength and blood pressure, whereas the effect on muscle size and biomarkers failed to reach statistical significance and needs to be addressed in larger and longer studies. Moreover, BFR resistance training seems to be potentially safe, with beneficial impact on hemodynamic responses, but not on vascular function. Therefore, it may be provided as an additional exercise option to safely and effectively improve skeletal muscle functioning and general health. Nonetheless, the current study reveals a need for future studies to evaluate the current findings on larger samples with longer training duration. Thus, such training effect on different health parameters can be exaggerated and later translated into early phase of cardiac rehabilitation.

## Ethics Statement

The protocol was approved by the Republic of Slovenia National Medical Ethics Committee and was registered on ClinicalTrials.gov (Identifier: NCT03087292). The study was conducted in accordance with the Declaration of Helsinki and the American College of Sports Medicine guidelines for Use of Human Participants.

## Author Contributions

TK conceived the study design, recruited and consented the participants into the study, conducted the research, analyzed the data, performed the statistical analysis, interpreted the data, drafted the manuscript, and was responsible for final content. MN conducted the research, analyzed and interpreted the data, and drafted the manuscript. KT conducted the research and drafted the manuscript. VS interpreted the data and drafted the manuscript. BJ conceived the study design, analyzed and interpreted the data, and drafted the manuscript. All authors read, critically reviewed, and approved the final version of the manuscript.

## Conflict of Interest Statement

The authors declare that the research was conducted in the absence of any commercial or financial relationships that could be construed as a potential conflict of interest.

## Footnotes

1 https://www.dtu.ox.ac.uk/homacalculator/index.php
